# Novel Cold-Adapted Esterase MHlip from an Antarctic Soil Metagenome

**DOI:** 10.3390/biology2010177

**Published:** 2013-01-25

**Authors:** Renaud Berlemont, Olivier Jacquin, Maud Delsaute, Marcello La Salla, Jacques Georis, Fabienne Verté, Moreno Galleni, Pablo Power

**Affiliations:** 1 Laboratory of Biological Macromolecules, Centre for Protein Engineering, University of Liège, Institut de Chimie B6a, Liège, Sart-Tilman (4000), Belgium; E-Mails: ojacquin@ulg.ac.be (O.J.); Maud.Delsaute@doct.ulg.ac.be (M.D.); marlasa@alice.it (M.L.S.); mgalleni@ulg.ac.be (M.G.); ppower@ffyb.uba.ar (P.P.); 2 Department of Earth System Science & Department of Ecology and Evolutionary Biology, University of California Irvine, 3208 Croul Hall, 92697 Irvine CA, USA; 3 Puratos Group, Rue Bourrie 12, Andenne, Belgium; E-Mail: JGEO@puratos.com; 4 Puratos Group, Industrielaan 25, Groot-Bijgarden, Belgium; E-Mail: FVerte@puratos.com; 5 Department of Microbiology, Immunology and Biotechnology, School of Pharmacy and Biochemistry, University of Buenos Aires, Junin 956 (1113), Buenos Aires, Argentina

**Keywords:** α/β hydrolase, lipolytic enzymes, metagenomics, *p*-nitrophenyl-ester, cold-adaptation

## Abstract

An Antarctic soil metagenomic library was screened for lipolytic enzymes and allowed for the isolation of a new cytosolic esterase from the α/β hydrolase family 6, named MHlip. This enzyme is related to hypothetical genes coding esterases, aryl-esterases and peroxydases, among others. MHlip was produced, purified and its activity was determined. The substrate profile of MHlip reveals a high specificity for short *p*-nitrophenyl-esters. The apparent optimal activity of MHlip was measured for *p*-nitrophenyl-acetate, at 33 °C, in the pH range of 6–9. The MHlip thermal unfolding was investigated by spectrophotometric methods, highlighting a transition (Tm) at 50 °C. The biochemical characterization of this enzyme showed its adaptation to cold temperatures, even when it did not present evident signatures associated with cold-adapted proteins. Thus, MHlip adaptation to cold probably results from many discrete structural modifications, allowing the protein to remain active at low temperatures. Functional metagenomics is a powerful approach to isolate new enzymes with tailored biophysical properties (e.g., cold adaptation). In addition, beside the ever growing amount of sequenced DNA, the functional characterization of new catalysts derived from environment is still required, especially for poorly characterized protein families like α/β hydrolases.

## 1. Introduction

Lipases and esterases are enzymes active towards different substrates. Some of these enzymes are used to access carbon sources (e.g., cinnamoyl esterase) [1], some of them are regarded as pathogenic factors [2], others are involved in biocide degradation [3], but many of them have as yet uncharacterized physiological functions. Because of the increasing amount of available sequenced genomes and the possibility to construct DNA libraries from uncultured microbial consortia, many new hypothetical enzymes, including lipases and esterases, are now accessible. This finding provides the possibility to isolate new biotechnologically relevant catalysts from extreme environments [4] and to test the robustness of hypotheses derived from cultivable microorganisms [5]. 

Cold-adapted enzymes from psychrophilic organisms are supposed to display higher activity at low and moderate temperature when compared to their mesophilic homologs. Such a high activity at low temperature is associated with higher thermal instability [6]. In cold-adapted enzymes the active site is regarded as the most heat labile structural element whereas some other parts of the proteins can remain correctly folded over a wider range of temperatures [7]. Often, when increasing the temperature, the temperature of inactivation does not correspond to the apparent melting temperature of the protein [6,8]. 

Many cold-adapted enzymes have been characterized; xylanase [9,10,11], cellulase [12], amylase [13,14,15] and lipase-esterase [16,17], among others. Their analysis reveals the molecular basis of protein adaptation to cold. The adaptation to low temperatures is thought to proceed by minor structural changes of the protein through modification of non-covalent interactions leading to an increase of the flexibility of crucial parts of the protein rather than to a global increase of the flexibility [8]. A decrease of the amount of proline and arginine residues and an increase of the amount of glycine are also assumed to contribute to the increase of the flexibility, in some cases [12]. In addition, a more accessible active site is considered as an adaptation to low temperature [18].

Interestingly, the metagenomic approach offers a unique opportunity for isolating new enzymes from uncultivated psychrophilic microorganisms [19,20]. Although few cold-adapted enzymes have been isolated using the metagenomic approach [21,22,23], little is known about their thermal stability. 

In this work a metagenomic library, constructed from an Antarctic soil sample, was partially screened for lipolytic activity. One clone displaying esterase activity was isolated. After production and purification, its adaptation to cold temperatures was analyzed regarding both the activity and the stability.

## 2. Results and Discussion

### 2.1. MHlip Isolation and Sequence Characterization

From the Antarctic soil metagenomic library [24], one clone producing a blue halo after one week incubation at 18 °C on SBA-tributyrin media was selected as a potential esterase/lipase producer. 

Complete DNA sequencing of the insert (3 kb) revealed a unique putative gene, named *mhlip* (Genbank access. number. GU550075). The gene encodes a 262 amino acids hypothetical protein with a theoretical molecular mass of 28,075 Da and a pI of 5.2. The encoded protein, MHlip, displayed 83% amino acid identity to a putative cytosolic α/β hydrolase derived from *Acidovorax delafieldii* 2AN (Acc. Numb. ZP-04765427). The sequence also showed significant identity with many other mesophilic proteins including the arylesterase from *Pseudomonas fluorescens* (PFE-ESTE 26% id., acc. no. P22862.4), the non-heme bromoperoxidase (BPOA2, 30% id., acc. no. P29715.3) and the non-heme chloroperoxidase (PRXC-STRAU, 29% id., acc. no. O31168.1) from *Streptomyces aureofaciens*, and with the crystallized esterase YTXM from *Bacillus subtilis* (id. 26%, acc. no. P23974). 

**Figure 1:**
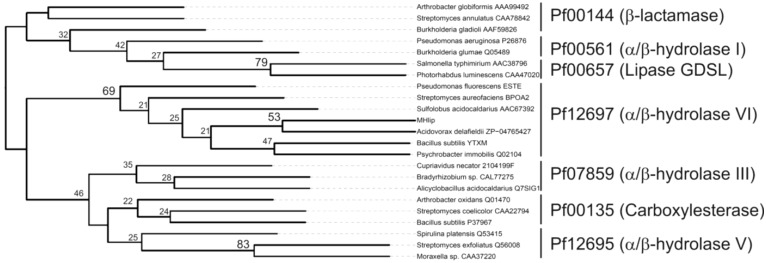
Unrooted neighbour-joining tree built with enzymes belonging to different families of esterases. The Jones-Tailor-Thornton method was used to compute distances [28]. Higher bootstrap values are displayed at the nodes and expressed as percentages of 1000 replicates. The Pfam numbers (names) are displayed on the right side of the tree.

We constructed a phylogenetic tree using the MHlip sequence and some sequences derived from various esterase-related families [25]. Next, Pfam numbers were assigned to each sequence using Pfam_scan (http://pfam.sanger.ac.uk/) [26] (Figure 1). MHlip was associated with the α/β hydrolase family 6 (Pf12697). This fold is observed in many enzymes having multiple functions (*i.e.*, transacetylase, lipase, hydroxynitrile lyase, methylesterase, peptidase, haloperoxidase, carboxylesterase) [27]. 

**Figure 2:**
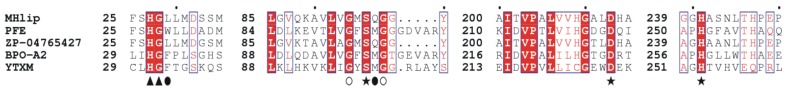
Sequences alignment of the MHlip enzyme and some related proteins. PFE-ESTE (P22862.4), aryl esterase from *P. fluorescens*; ZP-04765427, putative α/β hydrolase from *Acidovorax delafieldii*; BPO-A2 (P29715.3), bromoperoxidase from *Streptomyces aureofaciens* and YTXM (P23974.2), lipase from *Bacillus subtilis*. ★ residues forming the catalytic triad (Ser_96_, D_213_ and H_241_), ▲ residues forming the oxyanion hole involved in hydrogen bonding interactions, located upstream the active site (HG), ○ conserved residues involved in the formation of the GXSXG conserved pentapeptide containing the active-site serine, ● residues involved in the active site occlusion in PFE-ESTE [29].

Sequences alignment highlighted all the residues considered as involved in substrate binding and catalysis (Figure 2) [29]. In addition, residues W_29_ and M_95_ assumed to limit the active site of PFE-ESTE are displayed. These two residues limit the diffusion of large substrates in the catalytic cleft, independently of any lid structure. Thus resulting in an enzyme having high affinity for short substrates (*i.e.*, pNPA) [29].

### 2.2. Biochemical Characterization

The MHlip-encoding gene was PCR-amplified and introduced in the pET22b vector in order to express a C-terminal His-tagged MHlip. The recombinant protein was purified from the cytoplasmic fraction of transformed *E. coli* BL21(DE3) using Ni-NTA columns. 

**Figure 3:**
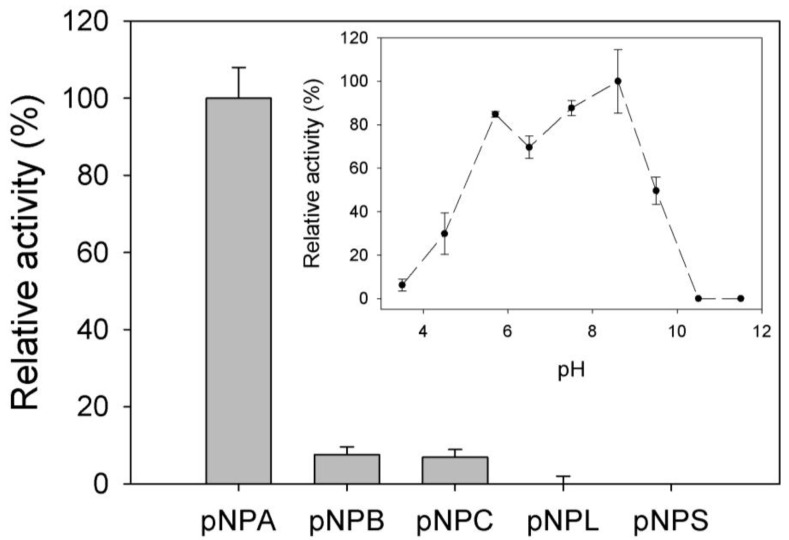
MHlip substrate specificity measured at pH 8 and influence of the pH on the MHlip activity (the inset).

Since MHlip was first isolated on tributyrin containing media, its activity was investigated on various *p*-nitrophenyl (pNP)-esters at pH 8.0 (Figure 3). MHlip is only active on small substrates such as pNPA; activity on pNPB is less than 5% of the pNPA hydrolysis and no activity was detected on substrates with longer acyl-chain lengths.

The pH-dependence of the MHlip activity on pNPA was investigated by monitoring the absorbance at 348 nm between pH 3.5 and 11.5, in a 20 mM pH-adjusted poly-buffer. MHlip was significantly active over a wide range of pH, from 4.5 to 9 (Figure 3). 

**Figure 4:**
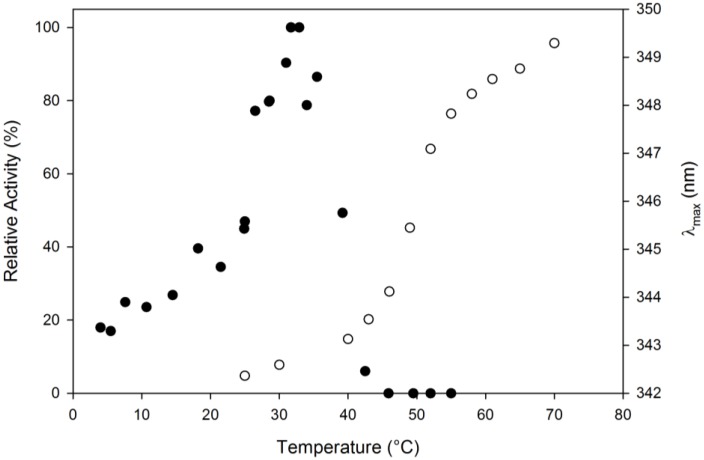
Influence of temperature on the MHlip activity (●). The enzyme was pre-incubated for 5 min at temperature ranging from 4 to 55 °C. Enzyme activity was determined under standard conditions on pNPA, at pH 8.0. The activity measured at 30 °C was taken as 100%. MHlip thermal denaturation following the maximum of fluorescence emission (λ_max_) in a thermal gradient ranging from 20 to 80 °C (○).

The thermal sensitivity of the MHlip activity towards pNPA was recorded at pH 8.0 in a thermal gradient ranging from 4 to 55 °C. The rate of pNPA hydrolysis rapidly increased from 4 to 35 °C and then quickly decreased to few percents of residual activity at 45 °C. Interestingly, MHlip remained significantly active when temperature decreased (20% at 4 °C) (Figure 4).

Kinetic parameters for pNPA hydrolysis were determined under initial rate conditions using a nonlinear regression analysis of the Michaelis–Menten equation. Hydrolysis was measured at 30 °C using pNPA as substrate at final concentrations ranging from 0 to 10 mM in 20 mM Tris-HCl (pH 8). For MHlip, the kinetic constants were: K_m _= 1.2~0.1 mM, k_cat _= 3.13.10^−2^~0.2.10^−2^ sec^−1^ and k_cat_/K_m _= 0.025 mM^−1^ sec^−1^.

### 2.3. Thermal Stability

MHlip fluorescence spectra recorded at 20 °C and 90 °C were compared. When MHlip was incubated at 80 °C its fluorescence emission spectrum, compared to the spectrum obtained at 20°C, revealed its unfolding. A bathochromic effect was observed when the temperature increased as the maximum fluorescence was shifted to higher wavelengths (from 338 to 350 nm). In order to determine the MHlip melting temperature, the fluorescence emission at 338 nm was recorded in a thermal gradient, from 20 to 90 °C (data not shown).

This approach appeared to be inappropriate since the increase of temperature caused a decrease of the fluorescence emission at 338 nm, with no clear transition. The determination of the apparent melting temperature (Tm) was eventually achieved by recording the wavelength of the maximum fluorescence emission (λ_max_) as a function of the temperature. At an excitation wavelength of 280 nm, a clear transition from a native to an unfolded state was observed at ~50 °C (Figure 4). 

## 3. Experimental Section

### 3.1. Metagenomic Library Construction and Screening

A previously described short-insert metagenomic library (average insert size of 5.1 kb) was used for the present work [19]. Screening for lipolytic activity was carried out by plating 3,000 recombinant *E. coli* clones on Spirit-Blue Agar (SBA) Media (BD-Difco, MD) containing chloramphenicol (12.5 µg/mL) and 1% emulsified Tributyrin (Sigma-Aldrich). The plates were incubated at 37 °C for 24 hours and kept at 18 °C for one week.

### 3.2. Expression and Purification

Forward primer (5’-GAGCACATATGCCTTTCGCGCA-3’) and the reverse primer (5’-GCTTCAGAGGCGCTCTCGAGCTTGTCGAGA-3’), containing *Nde*I and *Xho*I restriction sites, respectively (underlined), were used for PCR amplification of the full length MHlip-encoding gene. The obtained amplicon was ligated in an *Nde*I/*Xho*I double digested pET22b plasmid (Novagen, NJ) that allowed the production of a C_term_-6×HisTag fused protein. After confirmation of the sequence, the pET22b:MHlip was introduced in competent *E. coli* BL21(DE3) cells (Novagen). Transformed cells were cultivated at 18 °C in LB media containing ampicillin (100 µg/mL). Heterologous protein expression was carried out for four hours by adding 0.4 mM isopropyl-D-1-thiogalactopyranoside (IPTG) when the OD_600nm_ reached ~0.5. After centrifugation, the cell pellet was resuspended in 20 mM Tris-HCl (pH 8.0) and the cells were disrupted by sonication (3 cycles of 30 sec at amplitude of 10–12 µm). Proteins from the cytoplasmic fractions were recovered by centrifugation at 20,000 × g for 40 min, and MHlip was purified by affinity chromatography on a 5 mL Ni-NTA columns (GE Healthcare) in a linear gradient of imidazole (from 0 to 250 mM). Protein concentration was determined by the Bicinchoninic Acid (BCA)-protein quantitation assay (Pierce) using serum albumin as standard. Finally, 30 mg of pure protein was obtained per litre of culture. The purified protein was subjected to automatic Edman degradation for the determination of the N-terminal amino acid sequence on an Applied Biosystems 492 Protein Sequencer (Perkin Elmer, Waltham, MA, USA). The resulting sequence (MPFAH) was found to match the predicted one. The purified protein was used for further characterization.

### 3.3. Activity Assays

Lipase/esterase activity assays were carried out on *p*-nitrophenyl-esters (pNP-Acetate, pNPA; pNP-Butyrate, pNPB; pNP-Caprilate, pNPC; and pNP-Laurate, pNPL; all purchased from Sigma) by spectrophotometric methods. The assay mixture was prepared as follow: 940 µL of 20 mM Tris-HCl (pH 8.0), 10 µL of 10 mM *p-*nitrophenyl-ester in acetonitrile, and 50 µL of enzyme solution. The release of *p-*nitrophenol was followed taking into account a molar extinction coefficient at 405 nm (ε_405nm_) of 16,500 M^−1^.cm^−1^. 

The pH-dependence of the enzyme’s activity on pNPA, between pH 3.5 and 11.5, was investigated by monitoring the absorbance at 348 nm and replacing the Tris-HCl buffer by a 20 mM pH-adjusted polybuffer consisting of Tris, KCl, Na_2_HPO_4_, CH_3_COO.Na and dihydrogen citrate [30].

The thermal sensitivity of the MHlip activity toward pNPA was recorded as described above, over a temperature range between 4 and 55 °C.

### 3.4. Thermal Stability and Unfolding

In order to investigate the thermal stability of MHlip, the purified protein was submitted to increasing temperatures and the process of denaturation was followed by intrinsic fluorescence spectra. The spectrum of the native MHlip at 20 °C was recorded on a Perkin Elmer LS50B spectrofluorometer using a 1 cm cell path length. Maximum fluorescence emission was observed at a wavelength of 338 nm using an excitation wavelength of 280 nm. The Tm of the enzyme was determined by plotting the wavelength of the maximum emission (λ_max_), measured at temperatures ranging from 20 to 80 °C. 

## 4. Conclusions

The functional metagenomic is a powerful methodology to access the genetic material of environmental microorganisms. By the past, this approach has been used to isolated new enzymes from various environments [22,23,24,31,32,33]. Here, a new esterase form Antarctica is described. 

MHlip belongs to the α/β-hydrolase family 6 and is part of a large group of uncharacterized proteins. The physiological function of these enzymes has not been clearly elucidated [34]. In addition, MHlip displays significant identity (~30%) to various haloperoxidases and proteases. Although similarity between some esterases, oxidases and proteases was reported [29,35,36], MHlip does not show any detectable activity when tested for the conversion of the substrate phenol red to bromophenol blue [37] or azo-casein hydrolysis [38] (data not shown).

Among the tested substrates, the MHlip activity is highly specific for short esters such as *p*-nitrophenyl-acetate. Similar specificity for short substrates (e.g., pNPA) has been observed for the aryl-esterase derived from the goat rumen metagenome [39] and for the aryl-esterase from *Pseudomonas fluorescens* (PFE) [29]. Concerning PFE, such a high specificity is the consequence of a sterically hidden active site. Indeed, the catalytic cleft of PFE is delimited by W28 and M96 [29,40] whereas cumbersome residues, L29 and Q97, are observed in the corresponding positions of MHlip, and could thus have a similar effect. The kinetic parameters for the pNPA hydrolysis suggest that the MHlip primary function is not yet well understood. However, the measured Km for the pNPA hydrolysis (1.2 mM) is similar to some values obtained for previously characterized aryl-esterases, including the enzyme from Lactobacillus casei [41] and an aryl-esterase derived from the goat rumen metagenome [39].

**Figure 5:**
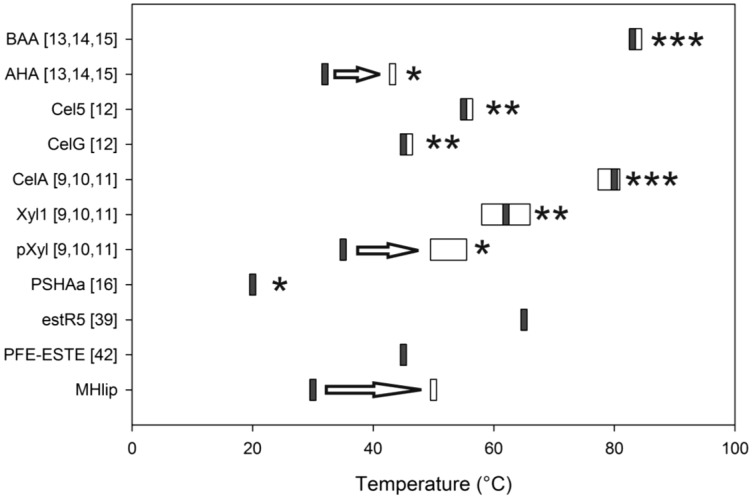
Shift in the observed optimal temperature of activity (gray rectangle) and the melting temperature (Tm, white rectangle) of some characterized proteins. *, ** and *** stand for protein considered psychrophilic, mesophilic and thermophilic in the corresponding references, respectively.

The characterization of purified MHlip reveals its adaptation to cold temperatures. Indeed, MHlip retains 20% activity at 4 °C. This is consistent with the temperature of the Antarctic soil sample used for the metagenomic library construction [24]. The MHlip activity increases from 4 to ~30 °C and is lost at higher temperatures. This suggests that our screening method, including a long incubation at cold temperature, is suitable for allowing the isolation of this highly thermo-sensitive protein. The aryl-esterases derived form the goat rumen metagenome (estR5) [39] and from *Pseudomonas fluorescens* (PFE) [42] are regarded as mesophilic homologs of MHlip since their optimal activity is observed at 60 and 45 °C, respectively (Figure 5).

The MHlip unfolding begins at 30 °C. At this temperature the MHlip activity is optimal. When increasing the temperature above 30 °C, the protein undergoes a partial denaturation and quickly loses its activity. Complete denaturation is apparently achieved at a temperature higher than 65 °C. Based on the fluorescence transition curve, the apparent T_m_ is determined to be 50 °C. 

The thorough characterization of cold active proteins unravels different ways for a protein to remain active at low temperatures [5,6,7,8,43]. Among these paths, increasing the length of the unstructured loop has been described in several cases [44,45]. However, MHlip sequence analysis does not reveal any extra stretches of amino acid.. Increasing the accessibility of the active site, as described for a cold adapted protease [18], was reported to increase the activity at low temperature. This is unlikely to occur here since MHlip may present cumbersome residues surrounding its active site. Moreover, the overall amino acid composition of MHlip is not significantly different from that of related proteins from mesophilic organisms. Nevertheless, this new enzyme, like most of the previously characterized cold adapted proteins, is an extremely heat labile enzyme.

Indeed, the MHlip’s loss of activity corresponds to the early stages of its unfolding. This suggests that the catalytic site is extremely thermo-sensitive compared to the overall protein structure. Shifts between the temperature at which proteins lose their activity and the unfolding temperature (Tm) have been recorded for many cold-adapted enzymes (Figure 5) [6,8,11,12,13,14]. Thus, even if no signature associated with cold-adapted protein can be observed, at the sequence level, we assume the MHlip adaptation to cold results from many discrete structural modifications that allow the protein to remain active when temperature is low. 
